# Equine Asthma Is Characterised by Severity‐Dependent Correlations Between Blood Neutrophil Cholesterol Content and NET Formation

**DOI:** 10.1002/eji.70072

**Published:** 2025-10-06

**Authors:** Lia K. Meiseberg, AhmedElmontaser Mergani, Julien Delarocque, Rabea Imker, Darleen Köhn, Dalanda Wanes, Marta C. Bonilla, Edwin J. A. Veldhuizen, Maren von Köckritz‐Blickwede, Bernhard Ohnesorge, Nicole de Buhr

**Affiliations:** ^1^ Clinic for Horses University of Veterinary Medicine Hannover Germany; ^2^ Institute of Biochemistry University of Veterinary Medicine Hannover Germany; ^3^ Research Center for Emerging Infections and Zoonoses (RIZ) University of Veterinary Medicine Hannover Germany; ^4^ Section of Immunology, Division of Infectious Diseases and Immunology, Department of Biomolecular Health Sciences, Faculty of Veterinary Medicine Utrecht University Utrecht The Netherlands

**Keywords:** anti‐neutrophil cytoplasmic antibodies (ANCAs), cholesterol, equine asthma (EA), horse, neutrophil extracellular traps (NETs)

## Abstract

Equine asthma (EA) is the most prevalent chronic lung disease in horses. Neutrophils are the main effector cells in severe EA. Neutrophil extracellular traps (NETs) have been described as contributors to severity in human asthma and chronic obstructive pulmonary disease. Thus, we aimed to investigate if NET‐related factors in equine neutrophils, blood and bronchoalveolar lavage fluid (BALF) allow us to differentiate EA severities and to identify NET‐related mechanistic insights in EA. We quantified NETs and NET‐related factors in the blood and BALF of eight healthy horses and 18 horses with differing EA severities. The proportion of activated cells in BALF increased with EA severity, accompanied by dysregulation of local NET regulators in severe EA. Furthermore, circulating anti‐neutrophil cytoplasmic antibodies (ANCAs = NET‐autoantibodies) were found elevated in severely diseased horses. In line with these findings, NET formation by circulating neutrophils was found to depend on the severity of EA. Finally, we analysed the cholesterol content of circulating neutrophils and identified an asthma‐severity‐dependent decrease in cellular cholesterol, which correlated with increased NET formation and hypoxia. Local and systemic modifications—particularly in neutrophil cellular cholesterol content—provide further insight into the partially understood pathogenesis of EA and point to a systemic cholesterol‐associated inflammatory component fuelling disease progression.

## Graphical abstract

Analysis of BALF and blood of asthmatic horses revealed local and systemic immunological variations related to NET formation and cholesterol homeostasis. Higher NET formation, lower cellular cholesterol and dysregulated NET regulators (e.g., eCATH, DNase, ANCAs) were associated with clinical disease severity. These results provide novel mechanistic insights into EA pathogenesis.

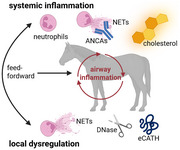

**Abbreviations**
AI: airway inflammation
AMP: antimicrobial peptide
ANCAs: anti‐neutrophil cytoplasmic antibodies
BALF: bronchoalveolar lavage fluid
CD: methyl‐*β*‐cyclodextrin
citH3: citrullinated histone 3
COPD: chronic obstructive pulmonary disease
EA: equine asthma
eCATH: equine cathelicidin
ETs: extracellular traps
HDL: high‐density lipoprotein
HPLC: high‐performance liquid chromatography
LDL: low‐density lipoprotein
mEA: mild–moderate EA
MPO: myeloperoxidase
NETs: neutrophil extracellular traps
sEA: severe EA

## 1 Introduction

Equine asthma syndrome is a complex, chronic, non‐infectious inflammatory disease of the equine lung. Clinical signs like chronic cough, dyspnoea, and exercise intolerance are caused by airway hyperresponsiveness, bronchoconstriction, elevated mucus production, and airway remodelling [1]. Barn and hay dust, fungal spores, inadequate circulation, and unidentified pasture allergens lead to disease progression [2]. Two EA phenotypes are distinguished by a worldwide consensus: mild–moderate (mEA) and severe EA (sEA) [1].

mEA is characterised by a mixed, neutrophilic, mastocytic or, in fewer cases, eosinophilic inflammation of the lung [2]. Persistent airway neutrophilia is a hallmark of sEA, showing similarities to neutrophilic late‐onset asthma of humans [3] and human chronic obstructive pulmonary disease (COPD) [4]. It has been proposed that EA can be employed as an animal model for the study of various human asthma phenotypes [5]. However, the underlying immune mechanisms reported for EA are inconsistent, with research focusing on the uniform sEA phenotype [6]. Nevertheless, there is clear evidence for a crucial role of neutrophils in disease progression, exacerbation, and chronicity [7].

During inflammatory processes, activated neutrophils release neutrophil extracellular traps (NETs) [8] with antipathogenic properties into the extracellular space. These scaffold‐like structures are composed of a DNA backbone and citrullinated histones that serve to enmesh pathogens and expose them to concentrated antimicrobial elements, including myeloperoxidase (MPO), and antimicrobial peptides (AMP), like the equine cathelicidins 1‐3 (eCATH1, eCATH2, and eCATH3) [8, 9]. NETs are described in infectious and non‐infectious diseases as well as in autoimmune diseases and can be induced by a variety of stimuli. They are, physiologically, strictly regulated through host DNases [10]. However, depending on multifactorial aspects, they can have beneficial as well as detrimental effects. Therefore, they are described as a “double‐edged sword”. Persisting NET structures and activated NET components in the airways can have detrimental effects on the host, triggering pathomechanisms such as direct cytotoxicity, neutrophil recruitment, autoimmunity, and chronic sterile inflammation [11, 12]. Moreover, unbalanced NETs were implicated in the pathophysiology of a variety of human pulmonary diseases, including asthma, cystic fibrosis (CF), and COPD, whereas small quantities of NETs can also be found in the lungs of healthy individuals [13, 14].

Increased NET formation correlated with disease severity in EA [15, 16]. Thus, it appears that NETs are involved in the pathogenesis of EA, but there is a paucity of knowledge about their contributions to this process. One key element influencing major cellular functions of neutrophils, including NET formation, is the cell‐cholesterol content [17, 18]. Interestingly, in CF patients, a reduction in plasma membrane cholesterol of peripheral circulating neutrophils was observed [17]. This is noteworthy, since CF is characterised by aberrant neutrophil migration, elevated NET formation and pulmonary injury, features also observed in EA [19]. Furthermore, hypoxia, a clinical condition observed in patients with severe lung disease like COPD, has the potential to influence cellular cholesterol synthesis, to increase neutrophil‐mediated damage, and, for example, to enhance anti‐neutrophil cytoplasmic antibody (ANCA)‐induced NET formation [20, 21]. ANCAs are auto‐antibodies against neutrophil granule and NET components that tend to hyperactivate neutrophils to form NETs [22]. ANCAs are connected to disease severity in CF and are found to be associated with severe asthma [23, 24].

This study aimed to identify whether neutrophil function, NET formation, the NET‐modulating factors eCATH and DNase activity, as well as cellular cholesterol content and ANCA concentrations, might contribute to the pathogenesis of EA. We further investigated cholesterol homeostasis‐associated parameters (total cholesterol, high‐density lipoprotein (HDL)‐ and low‐density lipoprotein (LDL)‐cholesterol) for their potential to serve as a non‐invasive diagnostic marker for the EA phenotypes. Additionally, we sought to explore potential associations between these immunological and metabolic parameters and relevant clinical features, to improve our understanding of disease mechanisms and to support the eventual development of new diagnostic and therapeutic strategies for EA.

## 2 Results

### 2.1 NETs and DNase Activity, as NET Modulating Factor, Vary Depending on the Severity of EA in BALF

**FIGURE 1:**
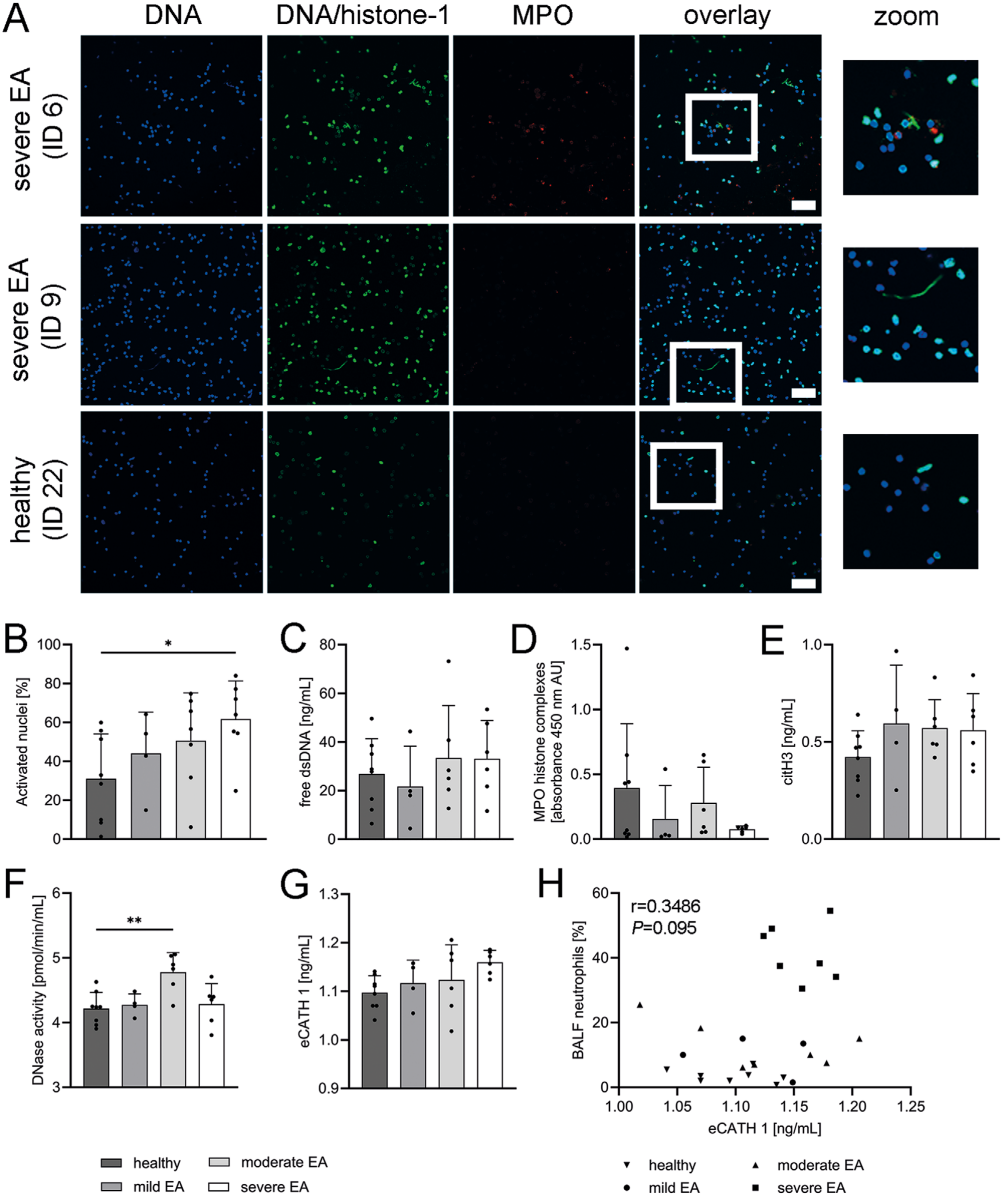
NETs and NET‐modulator analysis of BALF. (A) Fresh fixed cells in BALF were analysed for NET formation by confocal immunofluorescence microscopy. Representative images of NET‐activated nuclei (positive for DNA/histone‐1) from a healthy horse and two horses with severe equine asthma (sEA) are presented (scale bar = 50 µm). The zoom images show extracellular NET fibres and NET‐activated nuclei. (B) NETs were quantified in six randomly taken images per animal in eight healthy horses compared with four mildly, seven moderately, and seven severely asthmatic horses. NET markers ((C) free double‐stranded (ds)DNA, (D) myeloperoxidase (MPO)‐histone complexes, and (E) citrullinated histone3 (citH3)) were measured by ELISA. The NET modulator (F) DNase was evaluated quantitatively with an activity assay, and (G) the concentration of the equine cathelicidin1 (eCATH1) was determined by ELISA in cell‐free BALF supernatant available in eight healthy controls and horses classified as mild (*n* = 4), moderate (*n* = 6), and severe (*n* = 6) EA. (H) Spearman correlation of eCATH1 with proportion of neutrophils in BALF. Data are presented as mean ± SD, one‐way ANOVA followed by Dunnett's test for multiple comparisons (B–G), (*) *p *= 0.039; (**) *p *= 0.0031.

As EA is seen as a locally restricted disease, we initially investigated the occurrence of NETs in fresh bronchoalveolar lavage fluid (BALF) of horses suffering from varying asthma severities (mild (subclinical) (*n *= 4), moderate (*n *= 7), severe (*n *= 7)) compared with healthy controls (*n *= 8). Since chronic neutrophilic lung inflammation is a hallmark of sEA [1], and NETs were previously identified in BALF of horses with EA [16], we hypothesised that NETs would increase with disease severity. Indeed, using immunofluorescence confocal microscopy with a combination of nucleus‐staining and NET‐specific antibodies, we were able to identify increasing amounts of NET‐activated cells (for details see methods) in BALF with disease severity (Figure 1A,B). The DNA‐histone antibody used does not bind under conditions of normal chromatin condensation in neutrophils, but specifically stains cells exhibiting chromatin decondensation, as seen during NET formation [25]. Whereas neutrophils from healthy horses showed only 31.05% (±23.04) NET‐activated nuclei, this proportion increased with disease severity, reaching 61.72% (±19.62) in horses with severe asthma. Furthermore, NET fibres were observed more frequently in cases of sEA (Figure 1A, zoom picture). These findings support a link between disease severity and the extent of local NET formation.

Additionally, the occurrence of three selected NET markers in cell‐free BALF was measured via ELISA (Figure 1C–E): free double‐stranded DNA (dsDNA), MPO‐histone complexes, and citrullinated histone 3 (citH3). However, none of the NET markers was sufficient to distinguish disease severities, nor correlated with the amounts of activated cells. It has to be mentioned that cell‐free material is not a reliable marker for NETs, as extracellular DNA can also originate from other forms of cell death, making such substances in the cell‐free context inherently non‐specific.

Since NETs are tightly regulated by host DNases [26], DNase activity in BALF may have influenced both the detectability of NET markers and the persistence of chronic inflammation in the study horses [10]. Therefore, the DNase activity was quantitatively evaluated with a fluorometric activity assay, revealing elevated levels in horses with modEA, while activity was reduced in those with severe EA (Figure 1F).

In addition to DNase activity, a potent host factor known to modulate local NET dynamics is the antimicrobial peptide eCATH1. Similar to the human AMP LL‐37, eCATH1 may function as a neutrophil chemoattractant, as well as a NET inducer and stabiliser against nuclease degradation [27]. Here in our study, its concentration was found to be elevated in horses with sEA compared with healthy controls (Figure 1G, *p *= 0.061). Although eCATH1 in BALF did not show a significant increase, a clear trend is evident in relation to asthma severity, with a distinct cluster of sEA horses emerging in correlation with BALF neutrophil proportions (Figure 1H). Having identified phenotype‐specific differences in activated cells and the NET modulator nuclease, as well as eCATH, in BALF—both factors that may contribute to NET persistence in the lungs—we next investigated whether this could promote autoimmunity and the generation of ANCAs in EA.

### 2.2 ANCAs in Serum Increase in sEA

**FIGURE 2:**
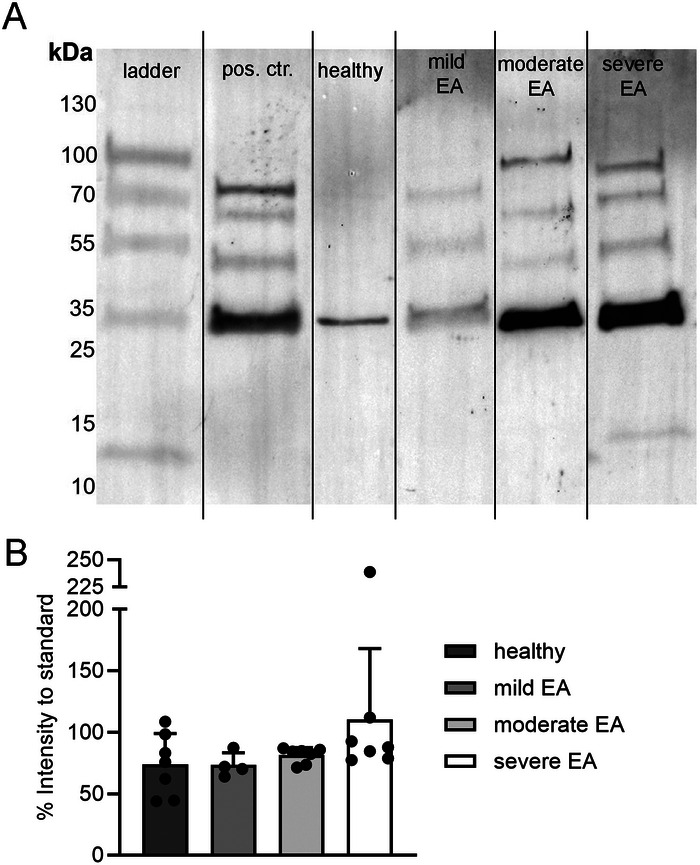
Detection of circulating anti‐neutrophil cytoplasmic antibodies (ANCAs) in sera. (A) A modified western blot was established in which the gel was loaded with NET components to detect free anti‐NET antibodies (IgG, IgA, IgM) in sera. The example image shows a customised protein ladder, a horse ANCA‐positive control serum (pos. ctr.), serum from a healthy control, and results from single horses with varying equine asthma (EA) phenotypes. Images of individual bands were cut from different gels to create the example figure. (B) The volume intensities of all bands for each serum were measured and calculated as a percentage of the positive control serum. Each dot represents one horse. Serum from eight healthy horses was compared with that of four horses with mild EA and seven classified as moderate and severe EA. Data are presented as mean ± SD, Mann–Whitney *U*‐tests: ns.

Based on the BALF results, we hypothesised that the dysregulated local NET formation in severely diseased animals would trigger autoimmunity against neutrophil‐derived proteins. In various autoimmune disorders, ANCAs have been linked to pathological side effects of NET formation and chronic inflammation [28]. Pulmonary involvement is a clinical feature of ANCA‐associated vasculitis [29]. To investigate the occurrence of ANCAs, we performed modified western blots in which we utilised digested NET proteins as antigens to detect circulating NET‐autoantibodies in the sera. The band sizes and patterns observed differed between individual animals and EA severities, indicating varying ANCAs in the study animals (example image Figure 2A). Interestingly, severely asthmatic horses showed higher total band intensities than all other groups; nevertheless, differences did not reach significance between healthy and sEA horses (Figure 2B, *p *= 0.064).

### 2.3 NET Formation by Peripheral Blood Neutrophils in Response to eCATH2 Increases with Disease Severity

**FIGURE 3:**
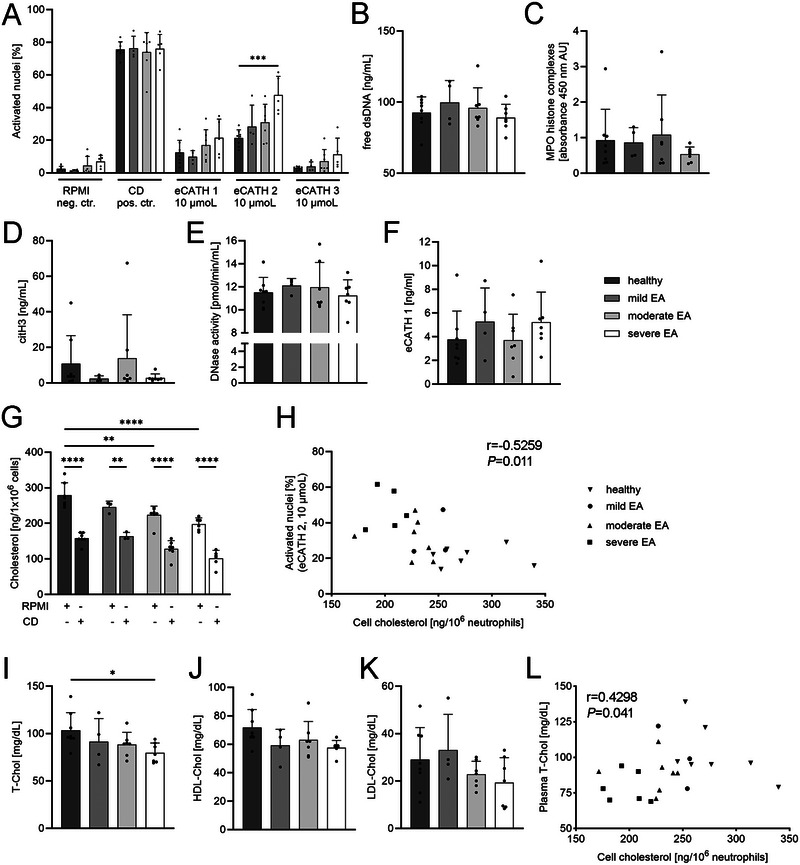
Analysis of NETs and associated parameters in blood. (A) Quantification of in vitro stimulated NETs from isolated blood neutrophils in immunofluorescence confocal microscopy images. Roswell Park Memorial Institute medium (RPMI) served as negative control (neg. ctr.), methyl‐*β*‐cyclodextrin (CD, 10 nM) as positive control (pos. ctr.), equine cathelicidin 2 (eCATH2, 10 µmoL) as species‐specific NET inducer. Circulating NET markers ((B) double‐stranded (ds)DNA, (C) MPO‐histone complexes, (D) citrullinated histone3 (citH3)) were measured by ELISA in serum. The NET modulators ((E) the NET‐degrading enzyme DNase and (F) the NET‐stabilising or inducing AMP eCATH1) were measured in serum by activity assay or ELISA, respectively. (G) Total cell‐cholesterol in isolated peripheral blood neutrophils was measured with high‐performance liquid chromatography after incubation with RPMI medium or treatment with CD, a potent cholesterol depletion agent. (H) Spearman correlation of NET formation of isolated blood‐derived neutrophils stimulated with eCATH2 (10 µmoL) (see also Figure 3B) and total neutrophil cell‐cholesterol. (I) Plasma concentration of total cholesterol (T‐Chol), (J) high‐density lipoprotein (HDL)‐, and (K) low‐density lipoprotein (LDL)‐cholesterol. (L) Spearman correlation of T‐Chol and total neutrophil cell‐cholesterol. Data are presented for eight healthy horses, and for four classified as mild EA, seven as moderate EA, and seven as severe EA. Data are presented as mean ± SD, one‐way ANOVA followed by Dunnett's test (A–F, I–K) or Tukey's test (G) for multiple comparisons, *p ≤ 0.05; **p ≤ 0.01; ***p ≤ 0.001; ****p ≤ 0.0001.

Since circulating ANCAs indicated variations in the systemic immune response of asthmatic horses, we hypothesised that the functionality of circulating blood neutrophils would also be affected in these animals. To investigate this, we isolated fresh peripheral blood neutrophils from the study horses using a density gradient, and we quantified ex vivo NET formation in response to eCATHs. As mentioned above, eCATHs are naturally occurring NET inducers in horses [30], and eCATH1 was found elevated in BALF in sEA correlating with BALF neutrophilia (Figure 1G,H). Interestingly, a difference in NET formation was observed when comparing the circulating neutrophils derived from different asthma groups after in vitro stimulation with equine cathelicidins (Figure 3A; Figure S1). Significant differences in NET formation in vitro were observed in sEA compared with healthy horses after incubation with eCATH2 (Figure 3A, *p *= 0.0004). These data indicated altered functions of circulating neutrophils in severe asthma, which is in line with the phenotype that elevated blood NETs have been observed in human patients with asthma and COPD [13].

However, in serum, neither the measured NET markers nor the analysed NET modulators (DNase activity and eCATH1) differed significantly between healthy horses or the EA phenotypes (Figure 3B–F). Moreover, we observed considerable inter‐individual variability within the groups. These observations pointed to intrinsic changes in circulating neutrophils, leading us to examine the correlation of NET‐forming capacity with cellular parameters of blood neutrophils in more detail.

### 2.4 Cholesterol of Blood Neutrophils Decreases with Disease Severity and Correlates with NET Formation

Considering the immense influence of cellular cholesterol on various neutrophil functions, including NET formation [18], we analysed the cholesterol concentrations of isolated peripheral blood neutrophils through high‐performance liquid chromatography (HPLC). Methyl‐*β*‐cyclodextrin (CD) was used as a control, which is well‐known to deplete cholesterol and induce NETs [31]. The total cell‐cholesterol was significantly different between the groups in both unstimulated neutrophils and CD‐treated cells (Figure 3G; *p ≤ *0.0001–0.0023). CD effectively depleted cholesterol from the cells in all horses, as previously shown for human neutrophils [18]. Importantly, neutrophils isolated from healthy horses exhibited a significantly higher total cell‐cholesterol content compared with moderate (*p *= 0.0016) and sEA (*p ≤* 0.0001). A similar trend was observable in mild EA, although it was not significant (*p *= 0.437).

As the neutrophils for the in vitro analysis of NET formation (Figure 3A) and the total cell‐cholesterol measurements derived from the same individual horse and the respective neutrophil isolation procedure, we were able to correlate the cellular cholesterol data with the NET formation. A significant negative correlation was observed between the cell‐cholesterol concentration and the NET formation (Figure 3H, *r* =−0.5259, *p *= 0.019), confirming that neutrophil cholesterol content affects NET formation in the study horses. The membrane stability is influenced by lower cholesterol and results in an easier stimulation and NET formation [18].

Based on this correlation, we hypothesised a link between systemic cholesterol homeostasis, NET formation, and equine asthma. To explore this further, we quantified cholesterol parameters in plasma to gain insights into cholesterol regulation and to evaluate their potential as easily measurable biomarkers for EA (Figure 3I–K). Plasma concentrations of total cholesterol decreased with disease severity and differed significantly between healthy and sEA (*p *= 0.0264). Again, a trend was also observable in subclinical horses (Figure 3I). LDL cholesterol decreased in moderate and sEA, although large inter‐individual variation is present (Figure 3K). Total cell‐cholesterol significantly correlated with total plasma cholesterol, indicating that there are systemic and cellular mechanisms influencing the cholesterol homeostasis (Figure 3L). To exclude liver disease, we analysed liver‐associated enzymes in all horses. There were no clinically relevant elevations above reference ranges, nor correlations between liver enzymes and cell‐cholesterol content within the groups (Figure S2).

### 2.5 Cholesterol Homeostasis and Hypoxia Are Connecting Local and Systemic Inflammatory Parameters

Chronic hypoxia is a known feature of EA and has been associated with disease severity and progression [32]. Beyond its clinical relevance, hypoxia also plays a regulatory role in cellular processes, including cholesterol homeostasis—by influencing cholesterol biosynthesis, uptake, and transport pathways [33, 34, 35]. Given these dual effects of hypoxia on both immune function and lipid regulation, we aimed to investigate whether hypoxia correlates with key local (e.g., NET formation in BALF neutrophils) and systemic (e.g., neutrophil cholesterol content, plasma cholesterol, circulating NET markers, and ANCAs) changes observed in EA. To this end, we used two established clinical parameters—arterial oxygen partial pressure (PaO_2_) and alveolar–arterial oxygen gradient (AaDO_2_)—as indicators of hypoxic status in our study horses.

**FIGURE 4:**
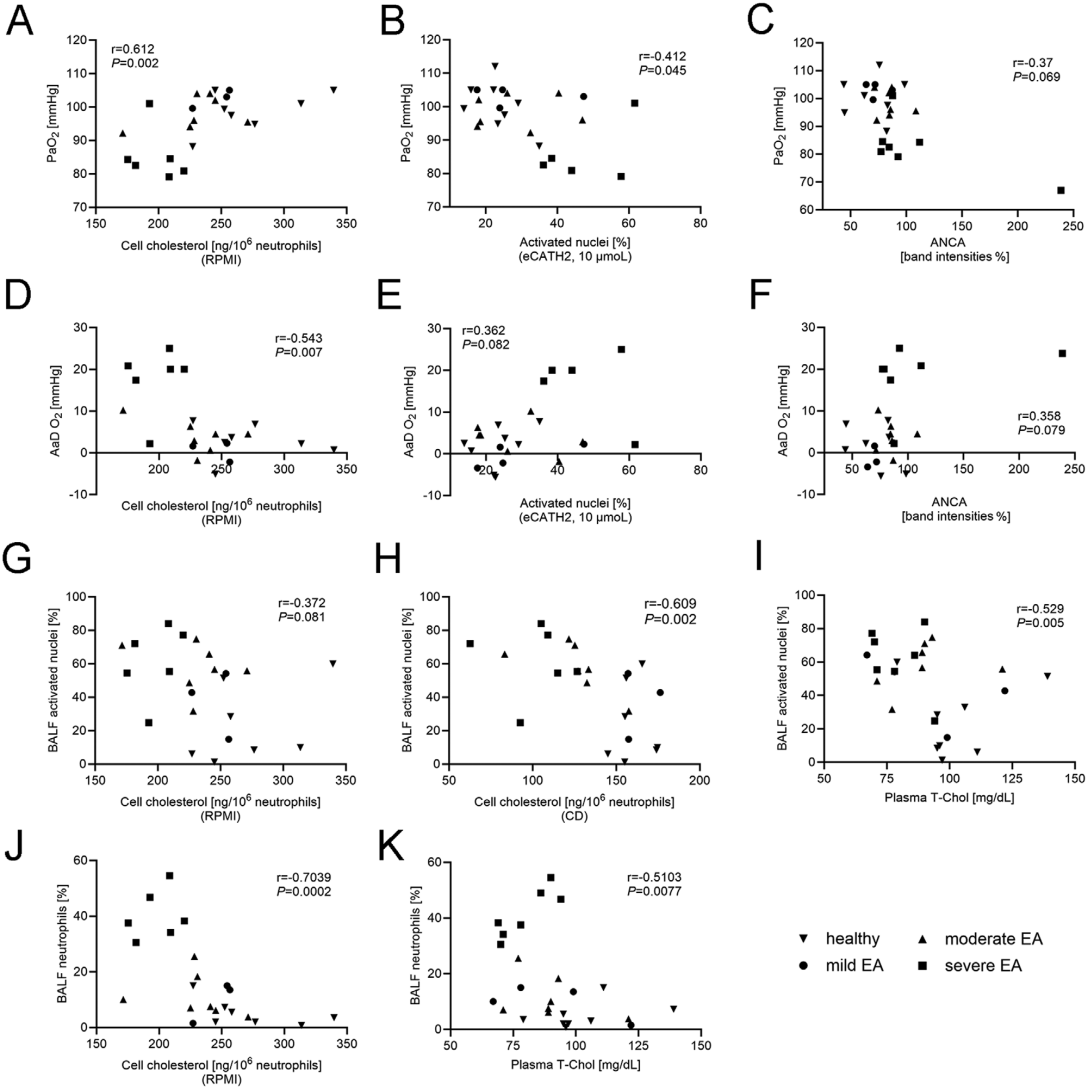
Spearman correlation analysis of clinical and NET‐related parameters. The arterial oxygen partial pressure (PaO_2_) was correlated with (A) the total neutrophil cell‐cholesterol of fresh isolated blood neutrophils incubated in Roswell Park Memorial Institute medium (RPMI), (B) the amounts of NETs after stimulation with equine cathelicidin 2 (eCATH2, 10 µmoL) in vitro, and (C) with the volume intensities of anti‐neutrophil cytoplasmic antibodies (ANCAs) in serum. (D–F) Correlations of the alveolar‐arterial oxygen gradient (AaDO_2_) with data analogous to PaO_2_. NETs in BALF were correlated with the total neutrophil cell‐cholesterol of fresh isolated blood neutrophils incubated in (G) RPMI and (H) methyl‐*β*‐cyclodextrin (CD, 10 nM) and (I) the total plasma cholesterol (T‐Chol) concentration. Correlation of proportion of BALF neutrophils with (J) the total neutrophil cell‐cholesterol of fresh isolated blood neutrophils incubated in RPMI and (K) the plasma total cholesterol concentration.

Indeed, Spearman correlation analysis revealed moderate to strong correlations between several variables (Figure 4): Significant correlations were found between hypoxia and neutrophil cell‐cholesterol (Figure 4A,D), as well as NET formation of isolated blood neutrophils (Figure 4B). Additional correlations linked NET abundance in BALF with neutrophil cholesterol concentration (Figure 4H) and with markers of systemic cholesterol homeostasis (Figure 4I). Furthermore, airway neutrophilia showed strong associations with both cellular and systemic cholesterol modifications (Figure 4J,K). These findings highlight hypoxia as a potential upstream modulator that links metabolic dysregulation to neutrophil activation and NET formation, suggesting an integrative mechanism contributing to chronic airway inflammation (AI) in equine asthma.

## 3 Discussion

**FIGURE 5:**
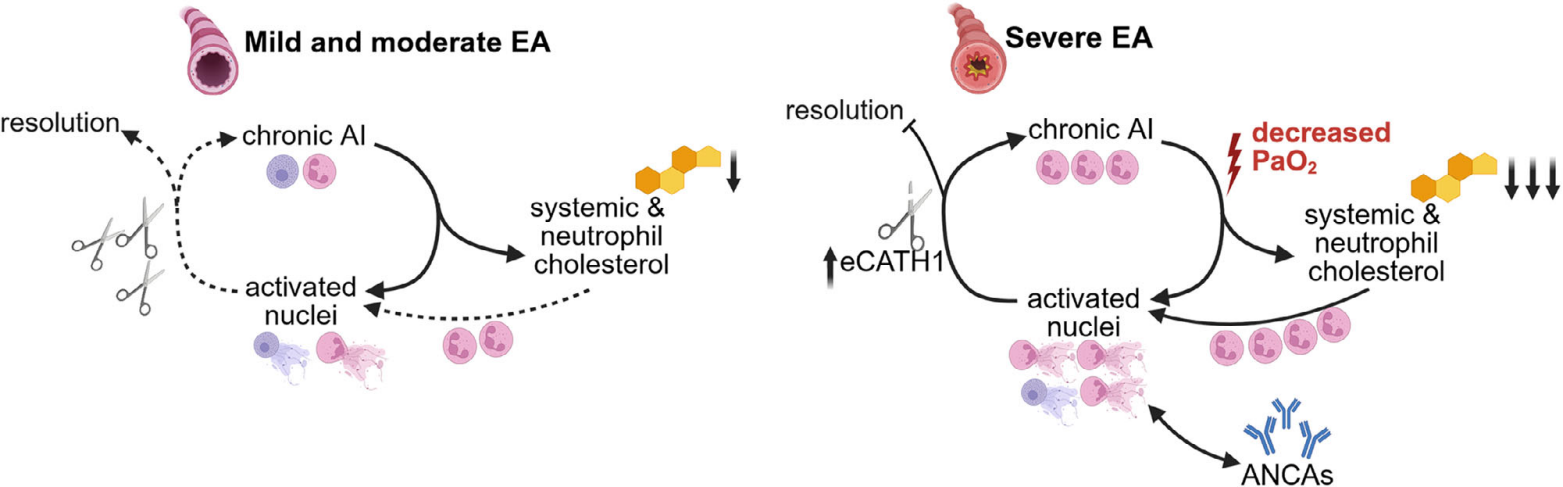
Schematic overview of hypothesised mechanisms during equine asthma pathogenesis. In mild and moderate EA (left side), neutrophils contribute to chronic airway inflammation (AI). AI is associated with mild variations in systemic cholesterol metabolism and may be accompanied by limited amounts of NET‐activated nuclei in the airways. A reduction in neutrophil cholesterol may lower the activation threshold of peripheral neutrophils, promoting increased neutrophil migration into the lungs and facilitating NET formation upon stimulation. However, NETs are subject to regulatory mechanisms, including the efficient production and activity of DNases, which facilitate their degradation and contribute to the resolution or attenuation of NET‐associated AI. In contrast, AI is dominated by high neutrophil numbers in severe EA (sEA, right side), associated with a low arterial oxygen partial pressure (PaO_2_), thus hypoxia. A marked decrease in neutrophil cholesterol leads to forced migration to the lungs. In the lungs, activity of the degrading enzyme DNase is insufficient to effectively control NET accumulation, and elevated equine cathelicidin 1 (eCATH 1) leads to NET stabilisation, induction, and chemoattraction of further neutrophils. The NET accumulation then triggers the production of anti‐neutrophil cytoplasmic antibodies (ANCAs). ANCAs can further contribute to the induction and stabilisation of NETs. These mechanisms drive the feed‐forward loop of chronic AI in sEA. The figure was created under license with biorender.com.

Reliable biomarkers for subclinical stages and progression of EA are still lacking, and the underlying disease mechanisms remain only partially understood. EA is a clinically relevant and widespread respiratory condition in horses, sharing key pathological and clinical features with human asthma and COPD [1, 2]. Our findings provide new insights into the pathogenesis of EA, which are summarised in a conceptual framework (Figure 5).

One key finding of this study was an increase in activated neutrophils and NET formation in horses with severe asthma, highlighting a likely mechanism by which NETs contribute to local AI. Considering this, we further examined factors known to modulate NET formation and persistence, including the antimicrobial peptide eCATH1 and DNase activity in BALF. Both parameters showed modifications in relation to disease severity, suggesting that an imbalance between NET production and clearance may play a role in sustaining AI in sEA.

Although eCATH1 concentration in BALF did not show a significant overall increase, its correlation with BALF neutrophil proportions revealed a distinct clustering of sEA horses (Figure 1H). Interestingly, previous studies have shown that neutrophils themselves do not release eCATH1 in horses [36], leaving the cellular source and regulatory mechanisms of eCATH1 expression in the asthmatic lung unclear. It can be hypothesised that, yet unidentified, factors drive the release of eCATH1 into the airways in a severity‐dependent manner. In our study, eCATH1‐3 was able to induce NET formation in circulating neutrophils from all horses examined in vitro (Figure 3A). Thus, neutrophils infiltrating the lungs—particularly in the high proportions observed in sEA—may be activated by cathelicidins alongside other unknown stimuli [8], contributing to the elevated presence of activated nuclei detected in the BALF (Figure 1A,B)

A second contributing factor appears to be a reduction in NET‐degrading capacity within the lung environment, depending on the EA phenotype. DNase activity as a NET‐regulating factor was significantly increased in moderate EA horses (Figure 1F), suggesting active NET turnover within the lungs at this disease stage. However, DNase activity declined again in sEA horses, supporting the hypothesis that NET regulation becomes impaired in advanced stages of EA, likely contributing to NET accumulation and sustained AI. This reduction could either reflect a general deficiency in DNase activity, as described in various autoimmune diseases [26, 37], or result from enzyme exhaustion, given that local NET formation in sEA may have been ongoing for an extended period.

Beyond the local factors influencing NET formation in the airways, our data point to a potential systemic component contributing to chronic inflammation in EA. The most severely and acutely diseased horse showed the highest amount of circulating ANCAs detected (Figure 2). This is of particular interest as in other immune‐mediated diseases, distinct ANCA subtypes are known to amplify inflammation by stabilising NET structures and hindering their clearance [38]. Moreover, certain ANCAs can actively induce further NET release [22], potentially perpetuating the cycle of neutrophil‐driven inflammation in EA. However, detection can be limited in disease remission and may be influenced by the methods used [39]. To elucidate ANCA occurrence in the airways and in asthmatic horses in exacerbation or remission, further studies are needed.

The observed local and systemic changes, in addition to the observed correlations, suggest that disruptions in cholesterol homeostasis are linked to disease severity (Figures 3 and 4). One explanation for the observed reduction in cholesterol content is the decreased oxygen availability in asthmatic horses. Hypoxia is known to alter lipid metabolism, including the regulation of cholesterol biosynthesis, transport, and efflux [33, 34, 35]. In the context of EA, a lower oxygen partial pressure could therefore exacerbate disruptions in cholesterol homeostasis, contributing to the metabolic and functional modifications observed in circulating neutrophils. Indeed, by correlating the arterial oxygen partial pressure with the cell‐cholesterol content, we observed a strong positive correlation (Figure 4A). This supports the hypothesis that increasing asthma severity—and the resulting hypoxia—may limit oxygen availability for cholesterol biosynthesis, thereby contributing to the reduced cellular cholesterol content observed in EA (Figure 5). Cholesterol synthesis is a process that is highly oxygen dependent [33]. Modifications in neutrophil cholesterol content were already known to modulate key neutrophil functions [17, 18, 40]. Although the design of this study does not allow us to establish direct causal relationships, the findings provide new insights into the pathogenesis of EA. For the first time, systemic changes in neutrophil function, NET formation capacity, cellular cholesterol content, and circulating cholesterol concentration have been identified in relation to disease severity, suggesting a potential metabolic and immunological component in the progression of sEA.

As a major transporter of cholesterol in the circulation, LDL plays a critical role in maintaining cellular cholesterol homeostasis, including that of immune cells such as neutrophils [41]. Disruptions in LDL concentrations may therefore influence both systemic lipid metabolism and immune function. A recently published human study measured LDL concentrations and reported that the risk of acute COPD exacerbations increased by more than 40% when comparing individuals in the lowest versus the highest LDL quartiles. The authors hypothesised that reduced plasma LDL cholesterol may reflect increased consumption during active disease processes [42]. Future research in horses should aim to more comprehensively assess cholesterol profiles—including plasma, blood cell, and tissue cholesterol—to better elucidate potential causal relationships between lipid metabolism and neutrophil function. One limitation of the present study was the restricted availability of blood samples, which limited the scope of lipid analyses in the horses. Detailed analysis of LDL cholesterol was not feasible, as triglyceride concentrations in many samples were near the lower detection limit of the assay used, thereby affecting the accuracy of LDL calculations. Despite these constraints, an interesting trend in the total cholesterol and LDL data was still apparent. As a future perspective, studies in horses could focus on refining lipid analysis methodologies and expanding sample sizes to validate these observations and to clarify the role of reduced plasma total cholesterol and LDL concentrations as potential biomarkers and modulators of disease progression in EA.

Given the observed reduction in neutrophil cholesterol content in EA horses (Figure 3G), we hypothesise the following mechanisms contributing to a worsening of the disease: first, a cholesterol‐dependent enhanced cell adhesion, as seen in CF [17]. Therefore, neutrophils could migrate faster into the lung. Second, a more rapid NET release after infiltration, driven by a NET‐promoting microenvironment and enhanced activation. Our data show that modifications in lipid metabolism of circulating neutrophils are linked to both increased NET formation and higher numbers of transmigrated neutrophils in BALF, in correlation with disease severity (Figure 3H; Figure 4G–K). This interpretation is further supported by transcriptomic data demonstrating enhanced neutrophil migration and NET‐forming potential in sEA [43].

Future studies should investigate whether neutrophils with reduced cholesterol content in horses with sEA are more readily activated by ANCAs, thereby amplifying the inflammatory feed‐forward loop and further linking the observed systemic and local modifications described in this study. However, the relationship between cholesterol and ANCAs has not yet been clarified.

In conclusion, by investigating neutrophil composition, NET formation, and NET‐modulating factors, we identified both local and systemic variations associated with EA in horses. Among these, cholesterol homeostasis—particularly neutrophil cholesterol content—appears as a distinguishing factor between the EA phenotypes. These findings indicate that EA is not merely a locally confined airway disease, but rather a complex systemic condition involving central metabolic modifications. In this context, analysis of neutrophil and plasma cholesterol profiles appears to hold promise as a tool for differentiating EA phenotypes. Larger studies will be required to establish reference ranges for neutrophil cholesterol, LDL, and HDL concentrations in horses and to validate their utility in phenotype classification.

## 4 Data Limitations and Perspectives

One limitation of the present study is the underrepresentation of horses with subclinical (mild) EA (*n* = 4). Another limitation concerns the methodology used for LDL analysis, as the lower detection limit for triglycerides restricted the accuracy of LDL measurements. To further clarify the role of cholesterol homeostasis in EA, future studies should employ more sensitive techniques, such as further lipoprotein‐fraction evaluation or HPLC‐MS lipidomics, and incorporate longitudinal designs to explore causal relationships between lipid metabolism, disease development, and progression. An important finding is that the number of activated cells detected in BALF exceeded the neutrophil proportions in both healthy and diseased horses [44], suggesting that extracellular trap formation by other cell types besides neutrophils may contribute to AI in EA. Future work should aim to characterise the various activated cell populations in BALF to understand the contribution of other cells, for example, inflammatory monocytes or mast cells, and their ET release in disease progression. Furthermore, a differentiation of sEA in exacerbation and remission in future studies could be beneficial to provide further insights into the underlying disease mechanisms.

## 5 Materials and Methods

### 5.1 Sample Collection: Blood and BALF

Samples were obtained from 26 client‐owned horses, which were examined at the Clinic for Horses of the University of Veterinary Medicine Hannover for diagnostic reasons.

Horses were grouped as healthy (*n *= 8), mild EA (*n *= 4), moderate EA (*n *= 7), and severe EA (*n *= 7) based on medical history, clinical examination, and BALF cytology [44]. Collection of blood was ethically approved by the State Office for Consumer Protection and Food Safety of Lower Saxony (LAVES) in accordance with the German Animal Welfare Law (reference: 33.9‐42502‐05‐21A626). Blood was drawn in the morning before the start of clinical examinations. After centrifugation (following manufacturer's instructions), aliquots of serum and plasma of EDTA‐, lithium‐heparin‐ and citrate‐anticoagulated blood were frozen in liquid nitrogen and stored at −80°C. Lithium‐heparin blood for neutrophil isolation was drawn 24 h after the end of examinations.

### 5.2 Collection and Analysis of Bronchoalveolar Lavage Fluid (BALF)

BALF was collected as described previously [44]. It was kept on ice, and cells, or supernatant, were fixed within a maximum of 90 min after collection. For staining of NET‐activated cells, 500 µL of BALF was added to poly‐L‐lysine (0.01%, Sigma Aldrich, P4707) coated glass‐slides (8 mm, VWR/Avantor, MENZCB00080RA120/CB00080RA12), priorly inserted in a 48‐well flat‐bottomed plate and centrifuged (250*g*, 4°C, 5 min). Cells were fixed with paraformaldehyde (PFA, final 4%; Science Services, E15710‐250). To collect cell‐free BALF, the remaining BALF was centrifuged twice (3000*g*, 4°C, 10 min). Supernatant was frozen in liquid nitrogen and stored in aliquots at −80°C.

### 5.3 Neutrophil Isolation

Equine neutrophils were isolated from fresh lithium‐heparinised blood with BioColl density gradient (Bio‐Sell, BS.L 6115, 1.077 g/mL) as described previously [8].

### 5.4 NET Induction Assay with Blood‐Derived Neutrophils from Asthmatic Compared with Healthy Horses

The NET induction assay with freshly isolated neutrophils was conducted as described previously [8] with slight modifications. After adding 2 × 10^5^ neutrophils and 100 µL stimulus per well, the plate was centrifuged (250*g*, room temperature, 5 min). The equine cathelicidins (eCATH1, eCATH2, eCATH3) [45] were diluted in Roswell Park Memorial Institute medium (RMPI) and used in final concentrations of 5 and 10 µM. After incubation, the plate was centrifuged (250*g*, room temperature, 5 min).

### 5.5 NET Induction Assay with Blood Neutrophils from Clinically Non‐Asthmatic Horses in Presence of BALF

Five NET induction assays were performed with isolated neutrophils from five clinically non‐asthmatic horses to investigate the impact of BALF from different phenotypes on neutrophils. Neutrophils were isolated and NETs stimulated in vitro as described above. RPMI was used as a negative control, whereas CD (final concentration 10 mM) and eCATH2 (final concentration 10 µM) were used as NET inducers. Neutrophils were incubated in the presence or absence of 100 µL BALF from horses included in this study. The BALF of horses with‐in the groups were chosen randomly to reach *n* = 5 for this experiment (healthy: IDs 23, 22, 19, 21, 4; mild EA: IDs 13 (2×), 11, 17, 20; moderate EA: IDs 1, 24, 16, 18, 8; and sEA: IDs 6, 5, 9, 10 and 7) [44]. Blood samples for neutrophil isolation for these assays were obtained by using fresh leftover blood from clinical diagnostics of patients from the Clinic for Horses. The first neutrophil donor horse was diagnosed with moderate asthma months after the assay was performed, and two out of five horses received non‐steroidal anti‐phlogistic medication (flunixin‐meglumine, 1.1 mg/kg BW) and antibiotics (amoxicillin 10 mg/kg BW and gentamicin 6.6 mg/kg BW) due to a tooth extraction and post‐colic surgery care.

### 5.6 Immunofluorescence Staining of NETs

Fixed cells from BALF and NET induction assays were co‐stained to quantify NET‐activated cells with DNA/histone1 and MPO as described previously [8]. Samples were stained for one hour with a monoclonal mouse IgG2 anti‐DNA/histone1 antibody (Millipore, MAB3864; 1.5 mg/mL) (1:2727) and a rabbit anti‐human MPO (Dako, A039829‐2, 4 mg/mL) diluted 1:374. Respective isotype controls were included: an IgG2α antibody from murine myeloma (Sigma, M5409‐1, 0.2 mg/mL) diluted 1:364 and an IgG antibody from rabbit serum (Sigma, I5006, 1.16 mg/mL) diluted 1:109. All antibodies were diluted in blocking buffer for one hour at room temperature. As a secondary antibody, a goat anti‐mouse Alexa 488 Plus antibody (Invitrogen, A32723, 2 mg/mL) and a goat anti‐rabbit IgG Alexa 633 antibody (Thermo Scientific, A21070, 2 mg/mL) were diluted 1:500 in blocking buffer, and samples were incubated for 1 h. After washing, a staining with aqueous Hoechst (Sigma, 14533‐100MG, 50 mg/mL) diluted 1:1000 in distilled water was conducted for 10 min. Slides were then embedded in Prolong Gold anti‐fade reagent (Sigma, P36930) and stored at 4°C in the dark.

### 5.7 NET Quantification with Confocal Microscopy

NET activation was visualised using a Leica TCS SP5 AOBS confocal inverted‐base fluorescence microscope with an HCX PL APO 40 × 0.75–1.25 oil immersion objective. Settings were adjusted with the respective isotype control. For every stimulus, or BAL fraction, respectively, six randomly taken images were analysed for ET‐activated cells. Therefore, three images per slide were taken. A cell was counted as positive when it showed a clear extracellular DNA/histone1 fibre off‐shoot or when the nuclei were positive for DNA/histone1 as NET marker, as described previously [8].

### 5.8 PicoGreen Assay

Free dsDNA in serum and BALF was measured using Quant‐iT PicoGreen dsDNA assay kit (Invitrogen, P11496) as described previously [46].

### 5.9 ELISA Analysis of BALF and Serum

According to the manufacturer's instructions, serum and BALF samples were evaluated with the following assays: a horse Cathelicidin1 (CATHL1) ELISA Kit (MyBioSource, MBS9374758) and a citrullinated histone3 (Clone 11D3) ELISA kit (Cayman, 501620). For quantification of MPO‐histone complexes, an indirect‐sandwich ELISA that has been established in the working group was performed [30].

### 5.10 DNase1 activity Assay

The activity of the NET‐degrading enzyme DNase1 was measured in serum and BALF using a DNase1 Activity Assay Kit (Fluorometric, Biovision, K429‐100) according to the manufacturer's instructions.

### 5.11 Cholesterol Measurements in Neutrophils and Plasma

Total cell cholesterol of isolated blood neutrophils was measured using HPLC. Peripheral blood neutrophils were processed directly after neutrophil isolation. In duplicates, 1 × 10^6^ cells per tube were treated either with RPMI or with CD (10 mM final concentration, C4555 Sigma Aldrich, Munich, Germany). Then, cells were incubated without closing the lid completely (5% CO_2_, 37°C, 3 h). After several washing steps with 1× PBS (lipopolysaccharide‐(LPS) free), the cell pellets were frozen at −20°C in high‐grade molecular water. The lipid isolation and cholesterol analysis were performed as previously described [47, 48].

Plasma total cholesterol (T‐Chol), HDL, and triglycerides, as well as the liver‐associated enzymes aspartate‐aminotransferase (AST), lactate dehydrogenase (LDH), alkaline phosphatase (ALP), and gamma‐glutamyl transferase (γ‐GT) were measured with a multi‐purpose automatic dry‐chemistry analyser (DRI‐CHEM NX500, Fujifilm) in lithium‐heparin plasma. LDL concentrations were calculated with the Friedewald equation.

### 5.12 Anti‐Neutrophil Cytoplasmic Antibodies (ANCAs)

For the detection of ANCAs, a western blot was performed with serum samples of diseased and healthy horses. To obtain isolated NETs, freshly isolated neutrophils were treated with CD (final 10 mM) in a 24‐well plate in a total concentration of 1 × 10^6^ cells/well as described above. After NET induction, 10 U/mL micrococcal nuclease was added per well, followed by incubation (5% CO_2_, 37°C, 20 min). Then, supernatants were pooled in a tube and centrifuged (300*g*, 4°C, 5 min). Isolated NETs were then aliquoted and frozen at −20°C. To quantify protein content of each aliquot, a Bradford Assay was performed following the manufacturer's protocol (BioRad, 500‐0006).

For each western blot, 25 µg NET proteins were used and mixed with 15 µL loading buffer (1 M Dithiothreitol diluted 1:100 in Lämmli buffer) and 15 µL PBS. Afterwards, protein samples were heated at 100°C for 5 min and then put on ice for 2 min before loading into the wells of a 4%–20% gradient gel. For every sample, one protein ladder was prepared to enable precise cutting, containing 15 µL of loading buffer, 11 µL of PBS, and 4 µL of protein ladder (PageRuler Plus Prestained Protein Ladder, 10–250 kDa, 26619). The gel was blotted onto transfer membranes (Carl Roth, 200T.1, pore size 0.45 µM) using a semi‐dry method. Afterwards, membranes were cut and pieces incubated overnight at 4°C on a roll shaker with different serum samples from healthy and diseased horses diluted 1:250 in blocking buffer (5% bovine serum albumin [BSA] in PBS). For each gel, an identical known ANCA‐positive serum sample was used as a control to verify signals in relation to this standard [30]. The next day, lanes were washed five times with 5 mL wash buffer (0.1% Tween 20 (Roth 9127.2) in 1× PBS) on a roll shaker. Afterwards, lanes were incubated, shaking with a polyclonal rabbit anti‐equine (IgG, IgM, and IgA), FITC, secondary antibody (Invitrogen, SA 1‐36092, 1.2 mg/mL), 1:300 diluted in blocking buffer (room temperature, 1 h). The lanes were then washed again as described. For readouts, a ChemiDoc MP system with BioRad Image Lab Touch Software (Version 2.4.0.03) was used with fluorescence settings for the serum samples and a calorimetric setting for the ladder. The bands were analysed using Image Lab Software (Bio‐Rad), and the percentage was calculated relative to a positive control running in each run.

### 5.13 Statistical Analysis

Sample size was determined by power analysis (*d*_Cohen_ = 2.52, *β* = 0.2, *α* = 0.05) based on NET formation in human asthmatics as published in 2016 [49] with the programming language R (version 4.3.0) [50]. Statistical analysis was performed with the programming language R and GraphPad Prism 10.4.0. Normal distribution was evaluated using the Shapiro–Wilk test. Group comparisons were performed with one‐way ANOVA followed by Dunnett's (Figures 1 and 3) or Tukey's (Figure 4) post hoc tests for pairwise comparisons or Mann–Whitney *U*‐tests (Figure 2). Correlations were described with Spearman's r. *p*‐values were adjusted for multiple comparisons. Figures were created using GraphPad Prism 10.4.0, and data are shown in mean ± SD. Statistical significance was set at a *p*‐value less than 0.05.

## Clinical Trial Registration

The study was registered by the State Office for Consumer Protection and Food Safety of Lower Saxony (LAVES) in accordance with the German Animal Welfare Law (reference: 33.9‐42502‐05‐21A626).

## Author Contributions

Conceptualisation: Nicole de Buhr and Bernhard Ohnesorge; methodology: Lia K. Meiseberg and Nicole de Buhr; validation: Nicole de Buhr and Lia K. Meiseberg; formal analysis: Lia K. Meiseberg, Julien Delarocque, AhmedElmontaser Mergani, Marta C. Bonilla, Dalanda Wanes, and Nicole de Buhr; investigation: Rabea Imker, Dalanda Wanes, Darleen Köhn, Marta C. Bonilla, AhmedElmontaser Mergani, and Bernhard Ohnesorge; software: Nicole de Buhr and Lia K. Meiseberg; resources: Edwin J. A. Veldhuizen and Maren von Köckritz‐Blickwede; data curation: Nicole de Buhr and Lia K. Meiseberg; writing—original draft preparation: Nicole de Buhr and Lia K. Meiseberg; writing – review and editing: all authors; visualisation: Nicole de Buhr and Lia K. Meiseberg; supervision: Maren von Köckritz‐Blickwede, Bernhard Ohnesorge, and Nicole de Buhr; project administration: Nicole de Buhr; funding acquisition: Maren von Köckritz‐Blickwede, Bernhard Ohnesorge, and Nicole de Buhr. All authors have read and agreed to the published version of the manuscript.

## Ethics Approval and Patient Consent Statement

Samples were obtained from 26 client‐owned horses, which were examined at the Clinic for Horses of the University of Veterinary Medicine Hannover for diagnostic reasons. Collection of blood was ethically approved by the State Office for Consumer Protection and Food Safety of Lower Saxony (LAVES) in accordance with the German Animal Welfare Law (Reference: 33.9‐42502‐05‐21A626). Horse owners gave informed consent for the use of data and samples.

## Conflicts of Interest

The authors declare no conflicts of interest.

## Peer Review

The peer review history for this article is available at https://publons.com/publon/10.1002/eji.70072.

## Supporting information




**Supporting File**: eji70072‐sup‐0001‐SuppMat.docx.

## Data Availability

The data that support the findings of this study are available from the corresponding author upon reasonable request.
