# Supplementary material for: Equine Asthma Is Characterised by Severity‐Dependent Correlations Between Blood Neutrophil Cholesterol Content and NET Formation
Source: Eur J Immunol. 2025 Oct 6;55(10):e70072. doi: 10.1002/eji.70072 (PMC12501399; doi:10.1002/eji.70072)

**Supporting information**

**Supporting information Figure 1:** Representative overview of immunofluorescence microscopy images from main Figure 3A. NETs were induced in isolated blood neutrophils with different stimuli *in vitro*. The quantification is shown in Figure 3A. For each equine asthma (EA) phenotype one horse is presented (scale bar = 100 µm, blue: DNA, green: DNA/histone-1 complexes, red: myeloperoxidase (MPO)). (Abbr.: RPMI = Roswell Park Memorial Institute medium, CD = methyl-β-cyclodextrin, eCATH = equine cathelicidin)


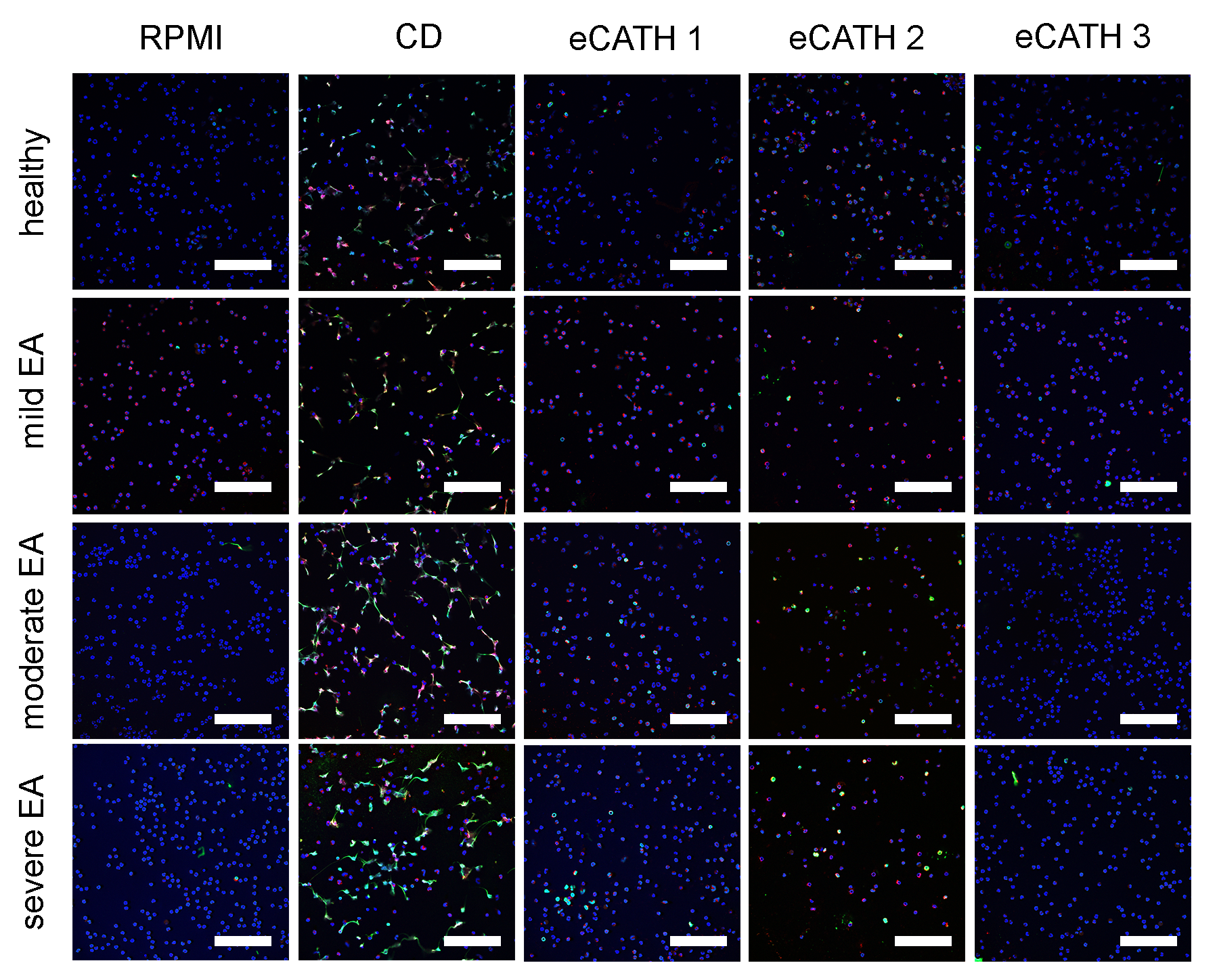


**Supporting information Figure 2:** Plasma concentrations of liver-associated enzymes and correlation to cellular cholesterol content. If within the measured ranges, the dotted lines represent the maximum reference value of the in-house laboratory. Single values above the references were not considered clinically relevant in horses without signs of liver disease if the other enzymes were within normal limits. Spearman correlation analysis revealed no significant correlations between liver-associated enzymes and neutrophil cell cholesterol of isolated unstimulated blood neutrophils (incubated in RPMI). Abbr.: LDH = lactate dehydrogenase, AST = aspartate-aminotransferase, ALP = alkaline phosphatase, γGT = gamma glutamyl transferase, RPMI = Roswell Park Memorial Institute medium. Data is given in mean ± SD. One-way ANOVA with Dunnett’s test for multiple comparisons: ns.


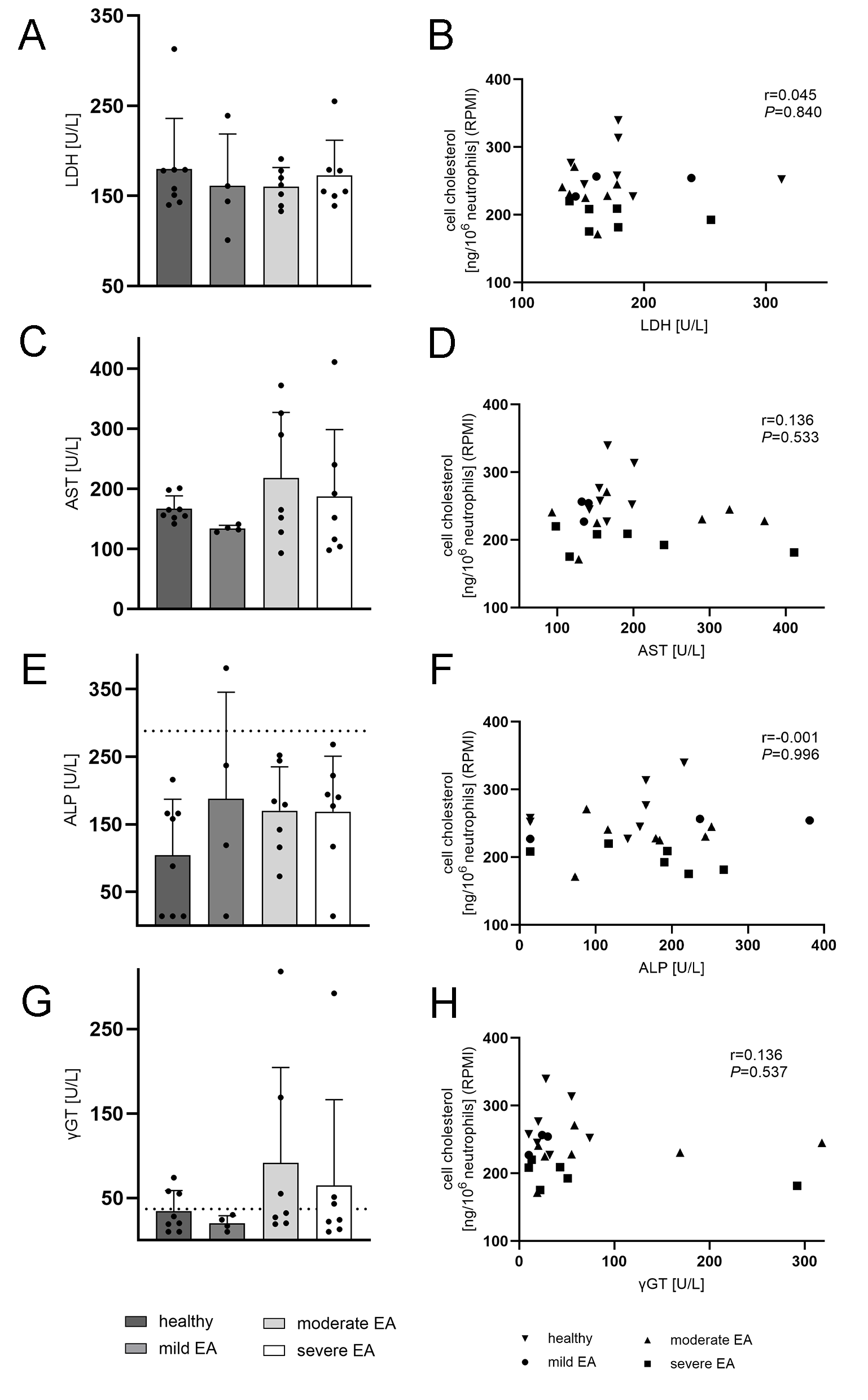


**Supporting information Figure 3:** NET formation of isolated blood-derived neutrophils from five non-asthmatic horses stimulated with BALF from the study horses. Neutrophils were incubated in RPMI (negative control) or stimulated with methyl-*β*-cyclodextrin (CD) or equine cathelicidin 2 (eCATH2). In the control (ctr.) no BALF was present to test the general function of the assay. In the RPMI, CD and eCATH 2 groups BALF from study horses was added as depicted to the control stimuli to investigate the effects of BALF on NET formation and stimuli function. Each colour indicates NET formation from the same clinically non-asthmatic donor horse. Data are presented as mean ± SD, unpaired Student’s t-tests, (*) *P=*0.034, (***) *P*=0.001


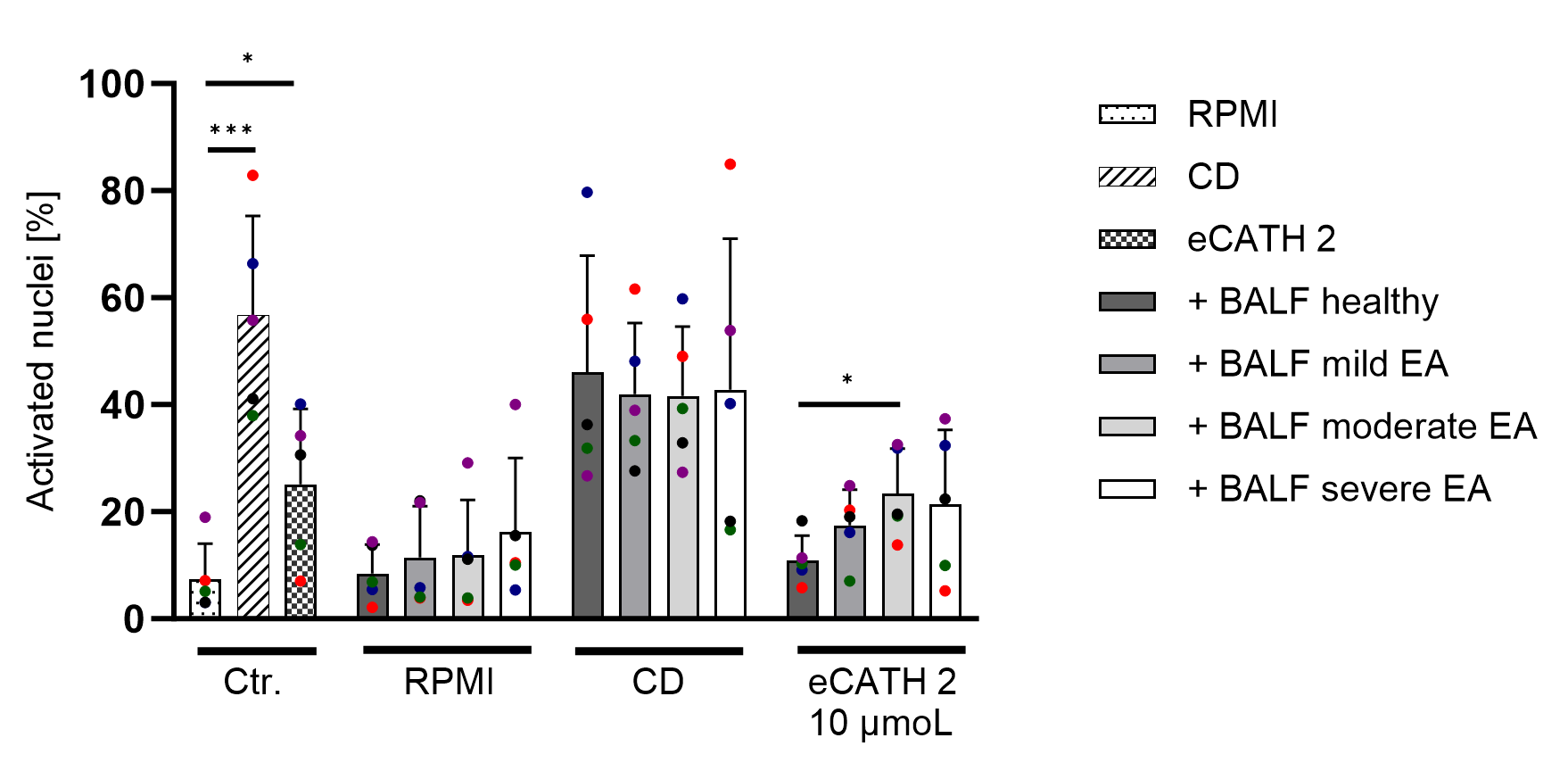

Supplement: Supplementary file 1 — Supporting File: eji70072‐sup‐0001‐SuppMat.docx. [file EJI-55-e70072-s001.docx]
